# The Validation and Determination of Empagliflozin Concentration in the Presence of Grapefruit Juice Using HPLC for Pharmacokinetic Applications

**DOI:** 10.3390/molecules29061236

**Published:** 2024-03-11

**Authors:** Wael Abu Dayyih, Zainab Zakaraya, Mohammad Hailat, Nafe M. Al-Tawarah, Sahem Alkharabsheh, Haya Khalid Nadher, Zeyad Hailat, Samia M. Alarman, Anas Khaleel, Riad Awad

**Affiliations:** 1 Faculty of Pharmacy, Mutah University, Al-Karak 61710, Jordan; erman.samia@yahoo.com; 2 Faculty of Pharmacy, Al-Ahliyya Amman University, Amman 19328, Jordan; z.zakaraya@ammanu.edu.jo; 3 Faculty of Pharmacy, Al-Zaytoonah University of Jordan, Amman 11733, Jordan; m.hailat@zuj.edu.jo; 4 Faculty of Allied Medical Sciences, Medical Laboratory Sciences, Mutah University, Al-Karak 61710, Jordan; nafitawa77@gmail.com (N.M.A.-T.); sahem@mutah.edu.jo (S.A.); 5 Faculty of Pharmacy and Medical Sciences, University of Petra, Amman 11196, Jordananas.khaleel@uop.edu.jo (A.K.); rawad@uop.edu.jo (R.A.); 6 Department of Information Systems, Yarmouk University, Irbid 21163, Jordan; zeyad.hailat@yu.edu.jo

**Keywords:** type 2 diabetes mellitus, sodium-glucose co-transporter inhibitor, (SGLT-2) inhibitor, empagliflozin, pharmacokinetics

## Abstract

Type 2 diabetes mellitus is a multifactorial disorder whose primary manifestation usually initiates with elevated blood sugar levels. Several antidiabetic agents are used to manage type 2 diabetes mellitus, of which empagliflozin is an oral sodium-glucose co-transporter (SGLT-2) inhibitor in the kidney. This research aims to develop and validate a simple analytical method for determining empagliflozin levels in biological fluid and to further evaluate grapefruit juice’s impact on empagliflozin pharmacokinetics in rats. High-Performance Liquid Chromatography (HPLC) was used to establish a simple, rapid, and accurate method for determining empagliflozin levels in rat plasma, in the presence of grapefruit juice. Four groups of rats (*n* = 10 rats in each) were used in the preclinical study. Group A (healthy rats) received empagliflozin alone; Group B (healthy rats) received empagliflozin with grapefruit; Group C (diabetic rats) received empagliflozin with grapefruit; and Group D (healthy, negative control) received no medication. The rats (*n* = 10) were given grapefruit juice instead of water for seven days before receiving the empagliflozin dose (0.16 mg/kg). Some pharmacokinetic parameters for each group were determined. The maximum plasma concentration (C_max_) and area under the curve (AUC) of empagliflozin in Group A without grapefruit intake were 730 ng/mL and 9264.6 ng × h/mL, respectively, with T_max_ (2 h). In Group B, C_max_ was 1907 ng/mL and AUC was 10,290.75 ng × h/mL in the presence of grapefruit, with T_max_ (1 h); whereas, in Group C, the C_max_ was 2936 ng/mL and AUC was 18657 ng × h/mL, with T_max_ (2 h). In conclusion, our results showed that the co-administration of grapefruit with empagliflozin should be cautiously monitored and avoided, in which grapefruit elevates the plasma level of empagliflozin. This may be attributed to the inhibition of the uridine enzyme in the grapefruit by hesperidin, naringin, and flavonoid.

## 1. Introduction

Diabetes mellitus (DM) is one of the most common chronic disorders, usually associated with elevated blood sugar concentrations [1]. DM is mainly caused by insufficient insulin production from the pancreas and low cell sensitivity to the naturally secreted insulin [2].

The inhibition of the sodium-glucose co-transporter (SGLT2) permits an increased excretion of renal glucose, leading to lowered blood glucose levels. SGLT2 regulates most renal glucose regeneration. The blood glucose decreases renal re-absorption and stimulates the kidney’s carrier protein, resulting in Urinary Glucose Exclusion [3]. The potency of its management is independent of insulin secretion and operation. This mechanism allows 1,3-biphosphoglycerate (BPG) to be combined with other antidiabetic therapies, giving the best management a complementary benefit. The SGLT2i function without insulin secretion is unaffected by β-cell depletion and insulin signaling desensitization [4]. 

Empagliflozin was the first antidiabetic drug to minimize cardiovascular and overall mortality in T2DM patients [5] with elevated cardiovascular risk, confirmed by new and significant clinical trials with SGLT2i in preventing hyperglycemia-induced risks [6]. No therapy has demonstrated comparable reductions in cardiovascular and overall mortality in T2DM patients with proven cardiovascular risk to date in either a dipeptidyl peptidase-4 (DPP-4) inhibitor or a glucagon-like peptide-1 (GLP-1) [7].

Pharmacokinetic or pharmacodynamic mechanisms can mediate drug–food interactions. A drug’s absorption difference may be clinically significant when considering its dosage and interactions with food [7]. For instance, slow-release theophylline formulations may differ in efficacy and can change upon consumption; the effectiveness of cyclosporine medicines may vary significantly based on the form taken and what is being co-administered with it. On the other hand, the effects of the medication on blood pressure or blood sugar levels in most patients are rarely clinically meaningful, as long as a rapid onset of those effects is not required [8].

The pharmacodynamics of certain drugs depend on the pre-existing chemistry of the body to generate the desired effect. When there is an incompatibility between a drug and the food taken by the patient, a pharmacodynamic interaction may occur [9], causing an antagonistic impact [8].

Due to the significant increase in grapefruit harvesting in the last decade, it has gained tremendous popularity [10]. Grapefruit enhances the bioavailability of some medications that interact with cytochromes (CYP450-3A4) [11]. The biological interest in grapefruit has encouraged chemical discovery, separation, and the characterization of several new substances [12].

This work aims to develop and validate a simple analytical method for determining empagliflozin levels in biological fluid using HPLC and also to evaluate grapefruit juice’s impact on empagliflozin pharmacokinetics in rats. We used a bio-analytical method for studying the effect of grapefruit on the pharmacokinetic parameters of empagliflozin, an SGLT2 inhibitor in type 2 diabetes mellitus in rats.

## 2. Results

### 2.1. Results of Validation

A partial method validation was performed to demonstrate the reliability of the stated HPLC method for determining empagliflozin and grapefruit concentrations in rat plasma using the parameters indicated in Table 1.

### 2.2. Accuracy and Precision

The method’s precision and accuracy were calculated by analyzing six samples with three replicates, each by two people on two days. The standard deviation (SD)-to-mean ratios were used to measure the relative standard deviation values (RSD) or CV %, expressed as percentages. For concentration and accuracy, the appropriate CV % limits, which should be less than 1.5, were determined. Furthermore, the agreed accuracy criterion of 85–115% for all concentrations was met.

The Quality Control Law (QCL) for empagliflozin analysis across six samples was performed. The analysis includes parameters such as the sample area, empagliflozin area, internal standard (IS) area, area ratio, actual concentration in nanograms per milliliter (ng/mL), theoretical concentration, accuracy, average accuracy, and relative standard deviation (RSD).

### 2.3. Absolute Recovery (Result of Matrix Effect)

The absolute recovery was calculated by measuring the fundamental empagliflozin peak region and an internal standard using an analytical method based on the plasma samples prepared for a drug concentration or an internal standard to ensure 100% recovery in the peak areas with pure standards. The degree to which the empagliflozin and the internal standard are recovered should be consistent, exact, and replicable. The accuracy measured at each level should not exceed 15% of the variance coefficient (percentage of CV). Absolute recovery (the result of the matrix effect) is illustrated in Table 2 below.

### 2.4. Grapefruit Effect on Empagliflozin Pharmacokinetics

Grapefruit juice is one of the most thoroughly researched dietary substances inhibiting CYP3A4 enteric metabolism.

### 2.5. Group A (Empagliflozin Alone)

**Figure 1:**
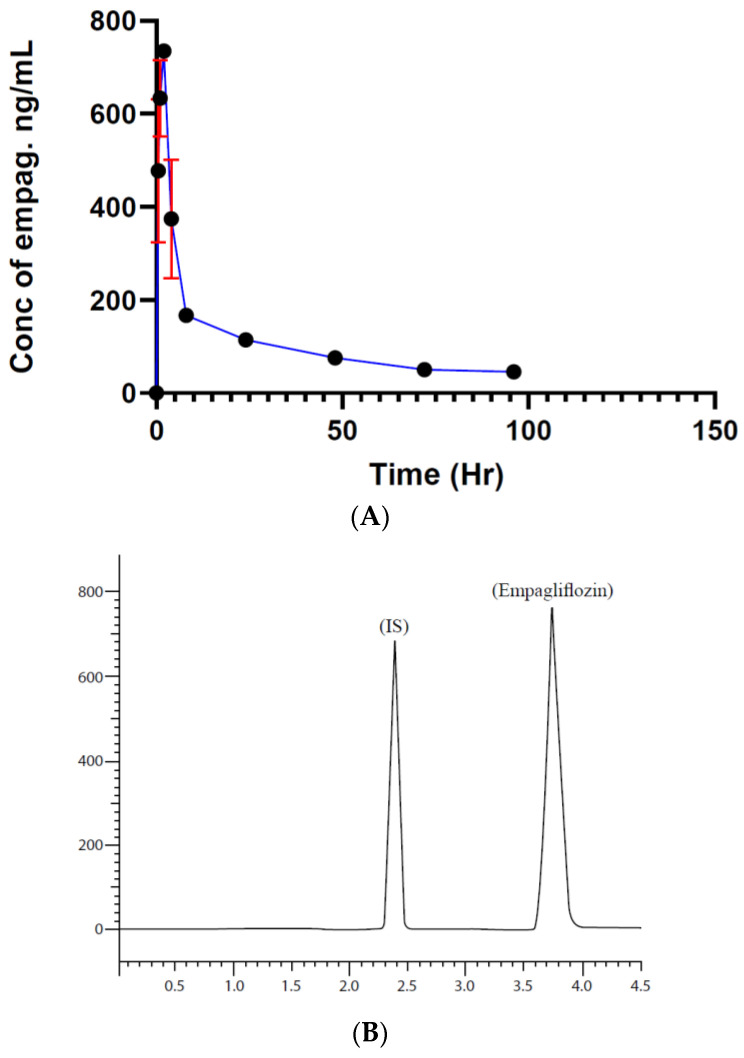
(**A**) Empagliflozin concentration vs. time plot. (*n* = 10), data ± SD, (**B**) HPLC chromatogram shows peaks and retention times for metformin (internal standard, IS) and empagliflozin.

According to the results shown in Figure 1, the maximum concentration of empagliflozin C_max_ 730 ng/mL was reached two hours after administration. A total of 26 h later, it gradually reached 49.2 ng/mL, the minimum concentration of empagliflozin. The area under the curve after 96 h (AUC_0–96_) was found to be 9264.6 ng × h/mL, as shown in Figure 1.

### 2.6. Group B (Empagliflozin and Grapefruit) in Normal Rats

**Figure 2:**
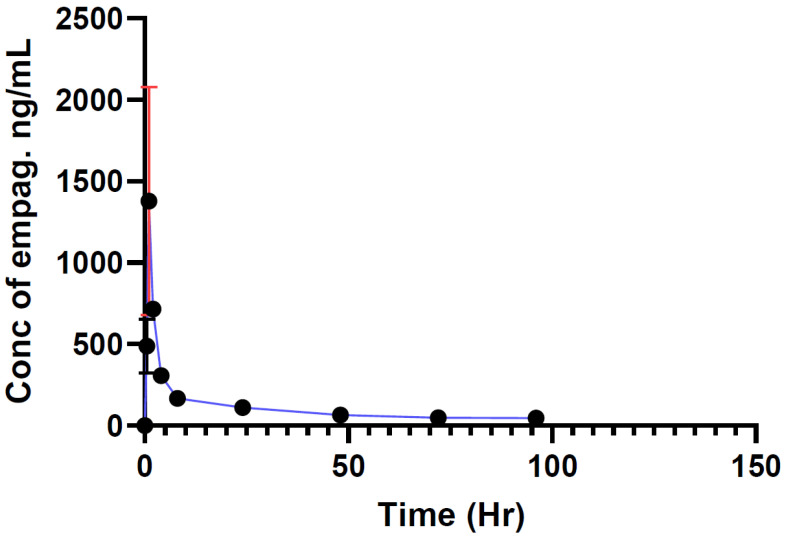
Empagliflozin concentration for normal rats treated with grapefruit vs. time (Group B, *n* = 10). Data ± SD.

According to the results shown below in Figure 2, the maximum concentration of empagliflozin C_max_ 1907 ng/mL was reached one hour after administration. A total of 26 h later, it gradually reached 45 ng/mL, recorded as the minimum concentration of empagliflozin. The area under the curve after 96 h (AUC_0–96_) was found to be 10,290.75 ng × h/mL, as shown in Figure 2.

### 2.7. Group C (Empagliflozin and Grapefruit) on Diabetes-Induced Rats

**Figure 3:**
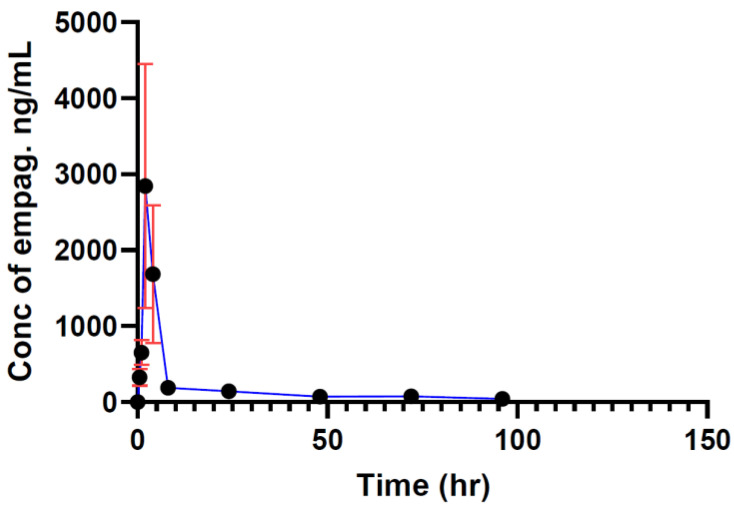
Empagliflozin concentration (grapefruit effect in diabetic rats, Group C) vs. time. *n* = 10, data ± SD.

According to the results below in Figure 3, the maximum concentration of empagliflozin C_max_ 2936 ng/mL was reached two hours after administration. A total of 96 h later, it reached 47 ng/mL gradually, accordingly recorded as the minimum concentration of empagliflozin to be achieved. The area under the curve after 96 h (AUC_0–96_) was 18,657 ng × h/mL, as shown in Figure 3.

Comparing empagliflozin pharmacokinetic parameters alone in normal rats in Group A and empagliflozin pharmacokinetic parameters in the presence of grapefruit on normal rats in Group B, the drug plasma level was increased in the presence of grapefruit. The T_max_ in Group B decreased to one hour and the C_max_ in Group B (1907 ng/mL) was increased due to the effect of grapefruit on the drug plasma level. The AUC for Group B (10,290.75 ng × h/mL) was also increased due to the impact of the grapefruit.

**Figure 4:**
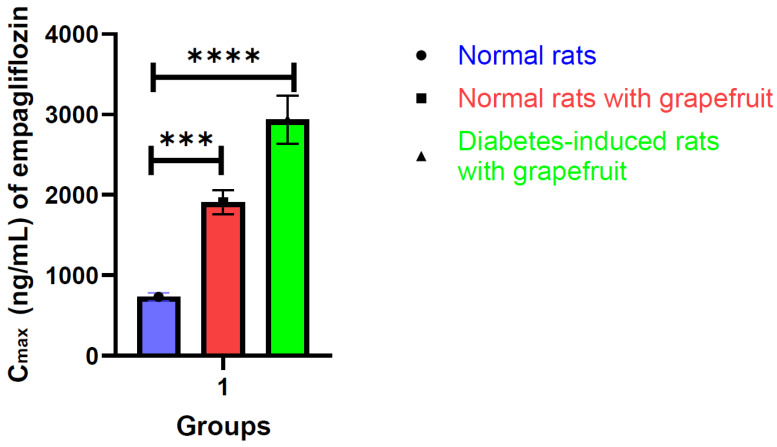
Histogram showing the average C_max_ of empagliflozin in normal rats, normal rats given grapefruit, and diabetes-induced rats given grapefruit (mean ± SD; ***, ****, indicates the *p*-value is less than or equal to 0.001, 0.0001, respectively).

**Figure 5:**
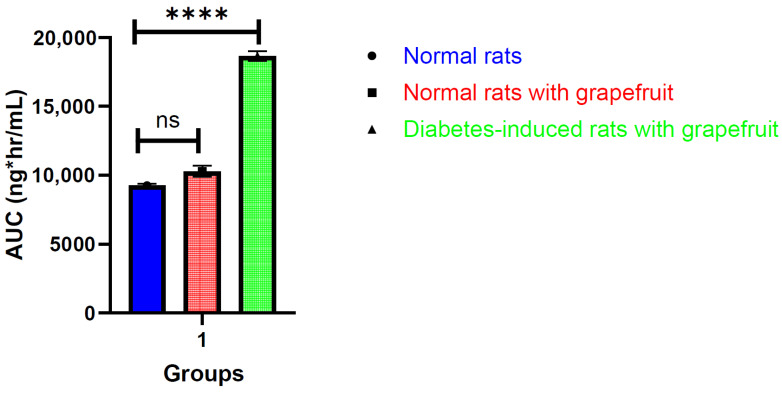
Histogram showing the AUC of empagliflozin in normal rats, normal rats given grapefruit, and diabetes-induced rats given grapefruit (mean ± SD; ****, indicates the *p*-value is less than or equal to 0.0001, respectively. ns: not significant).

Further comparing empagliflozin pharmacokinetic parameters with grapefruit on normal rats in Group B and with grapefruit in diabetes-induced rats in Group C, C_max_ was increased in induced rats in Group C. The T_max_ in Group C was increased in comparison to Group B, to become 2 h, the C_max_ and AUC in Group C were equal to 2936 ng/mL and 18,657 ng × h/mL, indicating that there is a highly significant increase in the C_max_ plasma and AUC as compared statistically, and as shown in Figure 4 and Figure 5.

## 3. Discussion

A partial, rapid, simple, and accurate method for determining empagliflozin levels in rat plasma has been developed using a High-Performance Liquid Chromatography–UV detector. Grapefruit administration affected the plasma empagliflozin level. When grapefruit was added to empagliflozin, the plasma Sitagliptin level increased dramatically compared to empagliflozin alone, especially in the DM-induced group.

Notably, the measured concentrations in the Quality Control Law (QCL) for empagliflozin analysis exhibit consistency with the theoretical values, indicating the robustness of the analytical method. The average accuracy of 103% reflects the precision and reliability of the analysis, while the low RSD of 0.30% highlights the minimal variability between measurements. These findings suggest a high level of confidence in the accuracy and precision of the empagliflozin analysis using the Quality Control Law approach.

The induced DM rats’ group, which takes empagliflozin with grapefruit, exhibited significant differences in the C_max_ and AUC compared to both normal groups, regardless of whether empagliflozin was administered with or without grapefruit. The C_max_ also increased in the presence of grapefruit in the normal group, but less than in the DM-induced group. From the results above, we can conclude that the plasma level of empagliflozin increases in the case of grapefruit intake in the induced group rather than the normal group because of the UGT2B7, UGT1A3, UGT1A8, and UGT1A9 enzyme inhibition, which may be associated with a change in the absorption profile of the drug, especially in the diabetic rats.

Empagliflozin is a substrate metabolized by uridine 50-diphosphate-glucuronosyltransferases that may affect empagliflozin’s pharmacokinetics and pharmacodynamics [13,14]. It is also a substrate for p-gp, BCRP, OATP1B3, and 1B1 in the intestine, partially responsible for empagliflozin’s active absorption [15,16]. Grapefruit juice is reported to have an inhibitory effect on p-gp efflux [17,18], a mechanism that might be involved in the absorption of this drug; this can partly explain the increase in the C_max_, accompanied by an increase in the rate of absorption, expressed as a shortening of the T_max_ [19]. The AUC could also be somewhat increased due to this reason as it increased slightly but significantly (*p* < 0.05).

As described earlier, empagliflozin metabolism is mediated through UGT isomers in the liver, where empagliflozin is also reported to have an inhibitory effect on them [20,21]. This explains the increase in the AUC and Kel in both groups treated with empagliflozin and grapefruit. Enzyme inhibition may result from the interaction of hesperidin and naringenin, which would result in a higher amount of the drug in the body for a longer time, explained by an increase in the AUC and a decrease in the Kel, as well as an increase in elimination half-lives [22].

This effect was of a higher magnitude in diabetic rats, possibly due to the changes in DM-related enzyme levels. The T_max_ of Group C was longer than that of Group B, possibly due to the exact reasons for the changed absorption pattern via transporters and the high effect of enzyme inhibition, which gave a greater extent of bioavailability, with a very long half-life. Our study has some limitations; for instance, only one model was used, and a more advanced model should be used in future studies to confirm the findings further.

When these results are compared to previously described results elsewhere [11] for sitagliptin (a competitive inhibitor of the dipeptidyl peptidase 4 (DPP-4) enzyme), several differences and similarities emerge, as shown in Table 3.

Both studies (this article and a previous one [11]) discovered that co-administering the medication with grapefruit juice increased drug levels (the C_max_ and AUC). However, the degree of increase differed between studies. The empagliflozin research revealed a more pronounced effect, particularly in the diabetic group. The sitagliptin study showed a statistically significant increase in the Cmax and AUC. However, the T_max_ decreased by half in the healthy group in the empagliflozin study, whereas the T_max_ did not shows a statistically significant increase in the sitagliptin study. Both studies indicate a clinically significant interaction, emphasizing the need to avoid grapefruit juice while taking these medications.

To summarize, the data indicate that giving empagliflozin with grapefruit juice increased drug levels (the C_max_ and AUC). However, the degree of increase differed amongst the treatment groups. The research indicated a more dramatic effect, particularly in the diabetic group. T_max_ was lowered by half in the healthy group. The study discovered a clinically significant interaction and suggested avoiding grapefruit juice when taking these medications. The summary of the research results is shown in Table 4.

More research may be needed to investigate the interaction mechanisms, individual patient variability in response, and the concentration of enzymes related to the metabolism and absorption of the empagliflozin drug.

## 4. Materials and Methods

Empagliflozin purity is 99.5% and was purchased from Sigma-Aldrich^®^ (Steinheim, Germany). Metformin HCL (99.5%) was obtained as a gift from Dar Al Dawa Pharmaceutical Company (Amman, Jordan). HPLC grade triethyl amine and HPLC grade methanol were from Fisher Scientific (Leicestershire, UK). Acetonitrile and Orthophosphate acid were purchased from Fisher Scientific (Leicestershire, UK). VWR International (Lutterworth, Leicestershire, UK) provided water (HiPerSolv CHROMANORM for HPLC) as the HPLC solvent. Streptozotocin (>95%; (bioXTra, London, UK), Lot # 18883-66-4) were purchased and used. All of the other chemicals were of reagent grade and used as received.

### 4.1. Instruments

An HPLC (FINNIGAN SURVEYOR) Liquid Chromatograph (Thermo Electron Corporation, San Jose, CA, USA) consists of a reciprocating quaternary gradient pump (LC Pump Plus) (Solvent delivery system pump), an auto-sampler (Auto-sampler Plus), a thermostatically controlled oven, a detector (UV-VIS Plus Detector), a communication bus module (CBM-20A), and a C18 column (Hypersil-Silica, C-18, 250 mm × 4.6 mm, particle size—5 µm, Thermo-Fisher, Cleveland, OH, USA). In addition, a Single-Pan Digital Balance (Sartorius) and an ultra-violet (UV) spectrophotometer (V530, version. 1.50.00, JASCO, Tokyo, Japan) controlled by Windows NT-based spectra manager were also used. A pH meter (model Sartorius 7110) was also used to measure pH. Centrifuge (M-24A, Boeco, Hamburg, Germany). Vortexes (Labinco, Breda, The Netherlands) and a sonicator (Elmasonic S100, Patterson, NJ, USA) were also used. The analysis was conducted at the University of Petra Pharmaceutical Centre’s Instrumental Laboratory.

Glassware such as volumetric flasks, funnels, beakers (Isolab, Wertheim, Germany Class A, DIN), pipettes (Isolab, Wertheim, Germany Class 2Aa, DIN), and micro-pipettes (Socoerx, ISBA S.A., Ecubleus, Switzerland) of 100 µL and 1000 µL capacity were used.

### 4.2. Animal Handling

The animals are described in detail in Table 5. The protocol of the study was approved by the ethical committee of the Scientific Research and Ethics Committee [SREC]–Faculty of Pharmacy/Mutah University (NO. SREC1132023, date 13 April 2023). The preclinical study was conducted at the Animal House of the Applied Science University. All the experiments were conducted as per the University of Petra and Applied Science Private University institutional guidelines on animal use, which adopt the Federation of European Laboratory Animal Science Association (FELASA) guidelines.

The rats were divided into four groups after marking each on its tail for identification; each group contained seven rats.

Group A: healthy—treated with empagliflozin 0.5 mL (0.16 mg/mL).

Group B: healthy—treated with grapefruit juice (10 mL/day) for four days. On the fourth day, they were treated with empagliflozin 0.5 mL (0.16 mg/mL).

Group C: Diabetic-induced group was treated with Streptozocin for three days at a dose of 45 mg; Streptozocin solution was prepared by dissolving 45 mg of Streptozocin in 12 mL of sodium citrate buffer and was mixed in a blender to obtain a homogenous solution of 3.75% (*w*/*v*), the concentration of Streptozocin was 3.75 mg/1 mL. As concerns the solution injected IP for each rat, after DM induction, the group was treated with grapefruit juice (10 mL/day) for four days (grapefruit juice will be given instead of water for seven days). On the fourth day, the group was treated with empagliflozin 0.5 mL (25 mg/150 mL). The Accu-Chek^®^ device was used for blood glucose measurements.

Group D: healthy—control no drug given (negative control).

### 4.3. Collection of Blood Samples

Blood samples were taken from the rats’ tails on the first day at defined time intervals, as follows: 0.5 h, 1 h, 2 h, 4 h, 6 h, 8 h, 24 h, 48 h, 72 h, and 96 h.

The samples were collected into an EDTA tube and centrifuged immediately at a speed of 5000 rounds per minute (RPM) for 10 min.

Plasma was obtained, placed into a labeled Eppendorf tube, and stored at −30 °C until analysis.

### 4.4. Sample Preparation

#### 4.4.1. Mobile Phase Preparation

Depending on previous studies [23] and trying several times to prepare the mobile phase, we found that the optimum buffer for the mixture of the prepared mobile phase was potassium dihydrophosphate buffer, which was designed by dissolving potassium dihydrophosphate salt (KH_2_PO_4_) in 1 L distilled water, then 1 mL of triethylamine added and, afterwards, the mixture was pH adjusted to 3.5, using orthophosphate. The mobile phase of acetonitrile–potassium dihydrophosphate buffer (50:50 *v*/*v*) was prepared. The flow rate was set to 0.75 mL/min, and the detection was achieved using a UV detector at 230 nm, with metformin as an internal standard. The time taken for the completion of the analysis was below five minutes. Metformin HCL and empagliflozin were identified using UV spectrum, peak purity, and retention times. All these chromatographic conditions were performed at ambient room temperature.

#### 4.4.2. Selection of Wavelength (λ) for the Chromatography

From the UV spectrum recorded on the UV–visible spectrophotometer, 230 nm was a suitable wavelength for detection. A solution containing 50 µg/mL of metformin HCL and empagliflozin in aqueous methanol (50%) was injected into the HPLC system and peak parameters were monitored at 230 nm.

#### 4.4.3. Preparation of Drug Solution

The rats were given 0.5 mL daily of 0.166 mg/mL empagliflozin, which means that we prepared a solution containing 25 mg of empagliflozin per 150 mL by milling an empagliflozin tablet (Jardiance^®^ 25 mg) and subsequently dissolving it in 150 mL of distilled water.

#### 4.4.4. Stock Working Solution Preparation of Empagliflozin

The empagliflozin stock solution was prepared by dissolving 100 mg of empagliflozin in 100 mL of methanol. The resulting solution contained 1 × 10^6^ ng/mL of empagliflozin.

#### 4.4.5. Stock Working Solution Preparation of Metformin

The metformin (IS) stock solution was prepared by dissolving 1000 mg of metformin in 100 mL of methanol, yielding a solution of 1 × 10^7^ ng/mL metformin.

#### 4.4.6. Preparation of Calibration Curve

To obtain ten spiked levels (for the calibration curve) in plasma, the stock solution was diluted in methanol to obtain the following calibration curve concentrations: 2.5, 5, 14, 16, 19, 30, 33, 45, 60, and 112.5 μg (10,000 ng/mL).

### 4.5. Method Validation

#### 4.5.1. Precision, Accuracy, and Absolute Recovery

The method’s accuracy and precision were calculated by testing six samples, each with three independent replicates, on two days. The standard deviation (SD)-to-mean ratios were used to calculate relative standard deviation values (RSD) or CV%, expressed as percentages. The CV% limits for concentration and accuracy were determined to be less than 1.5. Furthermore, the agreed-upon accuracy criterion of 85–115% for all concentrations was achieved.

The absolute recovery of empagliflozin was determined using plasma samples prepared for drug or internal standard concentrations. The accuracy of the recovery should be consistent, exact, and replicable, with a maximum accuracy of 15% of the variance coefficient (percentage of CV).

#### 4.5.2. Pharmacokinetic Analysis

Pharmacokinetic parameters were measured using the Winnonlin software V5.1, using a non-compartmental analysis (NCA) model. Estimations of the following parameters were made:

AUC_last_: area under the curve to 96 h. AUC INF: area under the curve to infinity. C_max_: maximum concentration of the drug in plasma. T_max_: time to achieve C_max_. t_1/2_: elimination half-life. Kel: elimination rate constant.

#### 4.5.3. Statistical Analysis

The statistical significance of the variable mean difference between the three groups, C_max_, T_max_, and AUC_last_, was calculated using GraphPad Prism software 10 (GraphPad Software Inc.; San Diego, CA, USA) and an independent *t*-test sample—significant *p*-value < 0.05.

## 5. Conclusions

To conclude, our results showed the complex effect of grapefruit consumption in high amounts on the pharmacokinetic parameters of empagliflozin, including both the absorption rate and the extent of bioavailability, and the final profile in diabetic rats may be the results of compensatory mechanisms associated with the administration of a high amount of this drug in rats. This might raise an alarm in taking this drug concurrently with grapefruit juice in humans.

Future studies are required to validate our results further. These involve using both in vitro and in vivo models, in which such findings may have severe complications and side effects on patients and may lead to a loss of drug activity.

## Figures and Tables

**Table 1:** Chromatographic condition summary.

Mobile phase composition	1 mL of triethylamine adjusted to pH 3.5 using orthophosphate, then (50:50 *v*/*v*) acetonitrile–potassium dihydrophosphate buffer.
Column type	C18 column (Hypersil-Silica, C-18, 250 mm × 4.6 mm, particle size—5 µm)
Wavelength	230 nm
Pump flow rate	0.75 mL/min
Auto-sampler temperature	25 °C
Column oven temperature	25 °C
Auto-sampler injection volume	20 μL
Retention Times (min)	
Metformin	2.4 min
Empagliflozin	3.7 min

**Table 2:** Absolute recovery (result of matrix effect, *n* = 7).

IS-Normalized Empagliflozin			
	Mean	SD	RSD%
QC_Low_	270.14	10.19	3.8
QC_High_	865	8.64	1.0
Absolute recovery	101.9	3.6	3.5
	100.6	0.9	0.9

**Table 3:** Comparisons between the effect of grapefruit juice co-administration on empagliflozin and sitagliptin pharmacokinetics [11].

Feature	Empagliflozin Study	Sitagliptin Study
Drug Dose	0.16 mg/kg	5.75 mg/kg
Rat Groups	Healthy (alone), Healthy (grapefruit), Diabetic (grapefruit), Negative Control	Control, Grapefruit Juice
Grapefruit Juice Administration	Seven days pre-dose	Grapefruit was given to the B group instead of drinking water two days before the study
Impact of Grapefruit Juice on the C_max_, AUC, and T_max_ compared with the controls	C_max_ increased, AUC increased, T_max_ decreased	C_max_ increased, AUC increased, T_max_ not changed
Grapefruit Juice Impact on the C_max_	Doubled in both healthy and diabetic groups	Significantly increased
Grapefruit Juice Impact on the AUC	Doubled in the healthy group, tripled in the diabetic group	Significantly increased
Grapefruit Juice Impact on the T_max_	Decreased by half in the healthy group	No significant increase
Conclusion	Avoid co-administration due to significant increase in drug levels	Drug–food interaction observed; avoid grapefruit juice at the same time.

**Table 4:** Pharmacokinetics summary of the four rat groups.

Group Number and Description	Pharmacokinetics Parameter Summary for Empagliflozin after Administration of Specific Treatment(s)
Group (A): healthy—treated with empagliflozin 0.5 mL (0.16 mg/mL) only	C_max_ (730 ng/mL), AUC (9264.6 ng × h/mL), T_max_ (2 h)
Group (B): healthy—treated with grapefruit juice (10 mL/day) for four days. On the fourth day, they were treated with empagliflozin 0.5 mL (0.16 mg/mL)	C_max_ (1907 ng/mL), AUC (10,290.75 ng × h/mL), T_max_ (1 h)
Group (C): A diabetic group was treated with grapefruit juice (10 mL/day) for four days (grapefruit juice replaced water for seven days). On the fourth day, the group was treated with 0.5 mL of empagliflozin (25 mg/150 mL).	C_max_ (2936 ng/mL), AUC (18,657 ng × h/mL), T_max_ (2 h)
Group (D): healthy (negative control) no drugs were given	no drugs were given

**Table 5:** Animal description.

Animal Species	Wisteria Rat
Weight	200 g
Number of animals	40
Gender	Males
Age	Eight weeks

## Data Availability

All the data are included in this article.
